# Analysis and visualisation of movement: an interdisciplinary review

**DOI:** 10.1186/s40462-015-0032-y

**Published:** 2015-03-10

**Authors:** Urška Demšar, Kevin Buchin, Francesca Cagnacci, Kamran Safi, Bettina Speckmann, Nico Van de Weghe, Daniel Weiskopf, Robert Weibel

**Affiliations:** School of Geography & Geosciences, University of St Andrews, Irvine Building, North Street, St Andrews, Fife, Scotland KY16 9AL UK; Department of Mathematics and Computer Science, Technical University Eindhoven, Eindhoven, The Netherlands; Biodiversity and Molecular Ecology Department, IASMA Research and Innovation Centre, Fondazione Edmund Mach, Trento, Italy; Department of Migration and Immuno-ecology, Max Planck Institute for Ornithology, Munich, Germany; Department of Biology, University of Konstanz, Konstanz, Germany; Department of Geography, Ghent University, Ghent, Belgium; Visualization Research Center, University of Stuttgart, Stuttgart, Germany; Department of Geography, University of Zurich, Zurich, Switzerland

**Keywords:** Movement ecology, Animal movement, Trajectories, Spatio-temporal analysis, Spatio-temporal visualisation, Geographic information science, Computational geometry, Visualisation, Visual analytics, Interdisciplinary developments

## Abstract

The processes that cause and influence movement are one of the main points of enquiry in movement ecology. However, ecology is not the only discipline interested in movement: a number of information sciences are specialising in analysis and visualisation of movement data. The recent explosion in availability and complexity of movement data has resulted in a call in ecology for new appropriate methods that would be able to take full advantage of the increasingly complex and growing data volume. One way in which this could be done is to form interdisciplinary collaborations between ecologists and experts from information sciences that analyse movement. In this paper we present an overview of new movement analysis and visualisation methodologies resulting from such an interdisciplinary research network: the European COST Action “MOVE - Knowledge Discovery from Moving Objects” (http://www.move-cost.info). This international network evolved over four years and brought together some 140 researchers from different disciplines: those that collect movement data (out of which the movement ecology was the largest represented group) and those that specialise in developing methods for analysis and visualisation of such data (represented in MOVE by computational geometry, geographic information science, visualisation and visual analytics). We present MOVE achievements and at the same time put them in ecological context by exploring relevant ecological themes to which MOVE studies do or potentially could contribute.

## Introduction

Understanding the processes that cause and influence movement is one of the challenges in ecological enquiry with consequences for other disciplines, such as biodiversity [1-3]. Movement ecology investigates fundamental questions about organismal movement, which include why, how, when and where the organisms move and how this process is linked to external factors [1,4]. This knowledge leads to understanding not only movement but also how and why animals use specific resources, how they interact with each other, with other species and with their environment and how they compete and reproduce - the key elements of evolutionary processes that determine survival and fitness [5]. Understanding the processes at the basis of movement will provide the link to population distribution and dynamics [6], which is essential to forecast the impact of human-caused environmental change and outline conservation strategies.

With recent advances in positional technology, ubiquitous accessibility and widespread use of global positioning devices, researchers are now able to track movement at unprecedented levels of spatial and temporal detail. Tracking devices have and will become smaller, cheaper and more accessible, new satellite tracking technologies are introduced, data download methodologies become more efficient, battery life increases, numbers and variety of sensors on tracking tags increase, and all this leads to more data being collected at even higher spatial and temporal resolutions. Thus, movement ecology transformed itself from its data-poor beginnings into a data-rich discipline, allowing to find new answers to the burning research questions in animal ecology. Additionally, due to the miniaturisation of devices, more and more species can be tracked, such as birds, small mammals or even insects, opening new possibilities for quantitative ecological investigation of species hitherto considered too small [7-11].

In addition, the diversity and integration of different sensors allow the focus of data collection to move from the observer to the observed individual. That is, by collecting information from various sensors (e.g. body temperature, heart rate, acceleration) and incorporating environmental information into movement analysis, it is now becoming possible to reconstruct an animal’s perception of the world. We can find out where the animal was, its activities in various places and at various times, how these places looked and felt like and how they might have impacted the behaviour. Such observations could eventually lead towards the animal becoming the sensor informing us about its environment [9,12,13].

The basis of all such investigations is positional information through time, which is currently mostly collected using some type of animal-borne GPS tracking device. Sometimes, data collection is also complemented with the conventional very high frequency (VHF) or satellite systems data (Argos system and ICARUS system- International Cooperation for Animal Research Using Space), but GPS data are becoming increasingly prevalent as locational information in movement studies [7,8,14].

Trajectory data, defined here as a discrete time series of measured locations, are collected at detailed temporal resolutions and on particular temporal schedules. Depending on sampling frequency and schedules, such data volume can be very large (long, densely sampled trajectories). Additional complexity is introduced with simultaneous collection of related information either directly from other sensors or derived from environmental data [5].

There are many challenges with trajectories, the most basic and urgent being to visualise and explore such data. New analytical and visualisation methods are necessary for this purpose [1,2]. While there has been little cross-disciplinary exchange so far, we believe that there is a significant potential in interdisciplinary connections between movement ecology and information sciences that analyse movement data. Such connections would facilitate and enhance the necessary new methodological developments to mutual benefit. They would provide information scientists with an opportunity to explore real problems and get access to real data, while movement ecologists would get support for challenging data issues from researchers who specialise in spatio-temporal data analysis and visualisation. New methodologies from such collaborations would be based on both data expertise and ecological domain knowledge, thus likely outperforming mono-disciplinary methods.

Trajectory data are commonly collected in many other disciplines where movements of objects are being observed (e.g. vehicle, vessel or plane trajectories for transportation, human trajectories in time geography, pedestrian trajectories for urban planning). Further, a set of disciplines across information sciences (geographic information science (GIScience), computational geometry, visualisation, visual analytics) specialises in analysis and visualisation of spatio-temporal data on movement, including trajectories [15]. Each of these disciplines has their own approaches to trajectory analysis and visualisation, but the underlying concepts are the same.

As an example of what can be achieved in such interdisciplinary collaborations, this paper presents a review of movement studies from the European COST Action IC0903 “Knowledge Discovery from Moving Objects (MOVE)” (http://www.move-cost.info/). A COST action is an international research network bringing together researchers from across Europe to collaborate on a common topic. The main objective of the MOVE action was to facilitate collaborations between researchers in disparate disciplines interested in movement, thus establishing a network of ICT researchers and domain specialists to enable the development of novel methods for movement analysis and visualisation. Researchers from various subdomains in computer and geographic information sciences joined domain specialists from a broad range of disciplines that collect movement data. The network was active in the period 11/2009 to 10/2013 and consisted of close to 140 individual researchers in 24 European countries. The network generated a wide range of activities including 6 network conferences; 13 workshops, including one in the Lorentz workshop series [16] and two in the Dagstuhl seminar series in computer science [17,18]; 5 PhD training schools; 7 data challenges; and 53 Short-Term Scientific Missions (short visits). The main activity was the formation of collaborative teams between researchers in information and communication technologies (ICT) and domain scientists, out of which movement ecologists were the most prominent group. These collaborations were active both formally (through funded joint research projects or PhD student co-supervision) and informally (through joint experiments and paper authoring) and are continuing after the end of the action.

On the ICT side, MOVE has inspired many novel methodological developments through the exposure of ICT researchers to real data and domain knowledge; [19] provides an example documenting this process of interdisciplinary collaboration. The aim of this review paper is to serve as the knowledge transfer vehicle into the opposite direction. By providing an overview of MOVE achievements and their potential relevance to movement ecology, we hope to contribute to increased recognition in the movement ecology community of the potential that collaborations with ICT researchers could bring.

This paper presents an overview of methods for analysis and visualisation of trajectory data that were developed in MOVE and were either 1) specifically aimed for movement ecology or 2) were not specifically developed for movement ecology, but have a potential to be used in this context, as they address similar topics in other application areas. We further list some related work from information sciences but outside MOVE which may be of interest to ecologists. To facilitate the interdisciplinary knowledge transfer, we put these studies in the context of four ecological themes that we were able to identify in MOVE collaborations. These themes are:

- **Theme 1:** Spatio-temporal dynamics of home ranges and utilisation distribution
- **Theme 2:** Identification of spatio-temporal patterns in movement
- **Theme 3:** Classification or identification of behaviour from movement data
- **Theme 4:** Linking movement data with environmental context

In the next section we provide a short introduction to each of these themes and discuss data analysis challenges inherent to each theme. In the second part of this review we then look at methods developed in MOVE and how each of these addresses one or more of the four ecological themes.

## ECOLOGICAL THEMES related to research in MOVE

### T1: Spatio-temporal dynamics of home ranges and utilisation distribution

**Utilisation distribution**, the probability of encountering an animal in a given location given the available locational data, is a formal way to quantify or represent animal home ranges [20-24], with the idea to identify areas that provide vital resources (food, protection, nest or bedding sites, support for mating encounters or group living), thus allowing maximisation of individual fitness. In practice, home range is still often derived as a certain probability contour of the utilisation distribution that represents the proportion of time spent by animals within this contour [25]. With the increased availability of detailed, and thus highly spatially and temporally autocorrelated data, the methodological limitations of most widely used methods of utilisation distribution quantification are becoming more and more apparent. For example, many methods are sensitive to sampling frequency, where with very high sampling the contours of the utilisation distribution hug the data increasingly tighter and therefore home range shrinks to the area of the measurement error around the trajectory. This means that current utilisation distribution methods may not properly represent the home range concept, as data are not invariant to sampling method and frequency and therefore violate the requirement of the statistical independence of the observations.

In this section we focus on a selection of methods for estimation of the utilisation distribution directly relevant to MOVE. Other reviews include [24,26-29]. A common approach is to employ kernel density estimators, which place a decay probability function on each observed location and sum these up into a surface [30]. The choice of density parameters (kernel function types, bandwidth size) is widely debated [25,26,31-33]. As in many statistical methods, there is a trade-off between bias and variance that needs to be taken into consideration with KDEs. While a higher level of smoothing increases precision, it also increases the bias. However as we collect data at increasingly higher sampling frequencies, the variance decreases – this means that less smoothing is necessary and bias is reduced [33].

One of the problems with standard kernel density estimators is that they rarely consider **the temporal dimension** and **sequentiality of points** in a trajectory. Linking spatial variation to fluctuations in size and distribution over time and between populations is sometimes done by determining space use separately in each temporal period (in different seasons, months or years) [34-36]. Alternatively, time can be included in definition of kernels, either in the calculation of the kernel [37] or by extending the kernels to cover trajectory segments between two consecutive points rather than individual points [38]. Among the latter the Brownian bridge kernels incorporate uncertainty in movement between two consecutive locations in the definition of the kernel [39-42]. Other approaches incorporate movement behaviour (such as periodicity of visits), landscape properties or memory into home range estimators [43,44].

Most of the existing temporal approaches to home range estimation in ecology are based on 2-D statistical tradition. Developments in MOVE however have **addressed the problem of the temporal dynamics of space use** from the perspective of analysis and visualisation of multidimensional spatio-temporal data resulting in conceptually new approaches discussed in the review section.

### T2: Identification of spatio-temporal patterns in movement

Movement of an individual organism is an interplay of four mechanistic components: its internal state, its motion capacity, its navigation capacity and the external factors [1]. The dynamic interaction of these components at various spatio-temporal scales is reflected in **spatio-temporal patterns in movement data**, which is why ecologists are particularly interested in identifying these patterns. We are searching for patterns from a variety of perspectives: within individuals or groups over time, between individuals, between groups, between populations, between species. In some cases, similarity in movement patterns is of interest, but so is identification of differences and relationships of these to both geographic space and time. In the following we present (non-exhaustive) lists of spatio-temporal pattern types of interest as well as of methods developed in movement ecology to identify these patterns in movement data.

One particular type of patterns is related to **routines**. These patterns are usually linked to temporal development of migration behaviour. **Migration** is a regular, seasonal pattern of movement that is strongly directional and seasonally reversible [45] and an obvious challenge is how to identify such routines or regular returns [46,47]. An unsolved problem in the study of migration is how learning affects migration journeys and migration ranges and how the range and/or route fidelity develop over time with an individual’s progressing age [48]. **Route fidelity** is a focus of many studies and can be investigated between individuals (birds flying in pairs), between the same individual at different times (consistency of the migration journey or route across seasons), or between individuals of co-existing species [49-52].

A related important question addresses **navigation in migration** [48]. How do orientation and long-distance navigation mechanisms influence the geometry of migration routes? What is the relationship between these mechanisms and migratory decisions (when to go, where to turn, what route to take)? Do the same navigational decisions occur at the same time and location in every migratory circle? What is the consistency of these decisions across individuals, groups and species? How do new migration routes evolve? Many studies are now exploring patterns in trajectory data in an attempt to answer these questions [48,53].

Another pattern type describes **dynamic interaction**, which is the inter-dependency of the movements of two or more individuals and is sometimes also called association, correlation or relative motion between two objects [54]. It can be investigated between individuals or groups of the same species, to see identify the frequency of individual encounters and patterns of avoidance, attraction, grouping or following [52,55]. Alternatively, patterns of interaction between co-occurring species can be of interest [51,56].

Identifying patterns from trajectory data requires a diverse set of methodologies. Temporal variability in movement can be explored through comparing long-term vs. short-term patterns, looking at seasonal patterns or presence of periodicity of varied lengths [47,51,55-59]. Route fidelity calls for geometric similarity analysis [50,52,53]. Migratory behaviour can be investigated through segmentation of trajectories at various spatio-temporal scales [46,47,53], where cross-scale analysis is of particular importance [60]. Interaction patterns can be identified using geometric approaches [61-63].

MOVE’s contribution to this theme is a series of **alternative methods for spatio-temporal pattern identification**. Computational geometry developed methods for median trajectories, segmentation, geometric similarity of trajectories and quantification of dynamic interaction. Spatio-temporal and attribute similarity of trajectories has been explored by GIScience through development of new data mining methods, such as geometric clustering, spatio-temporal clustering and clustering based on derived parameters of movement. Several contributions have also been made to cross-scale analysis.

### T3: Classification or identification of behaviour from movement data

Animal movement is linked to behavioural responses [64,65], so that specific behaviours correspond to different movement types. For example, foraging, escaping predators, sitting in the nest, soaring in search of prey, all intuitively correspond to different movement patterns. Two recent technological developments support new ways of analysing behaviour beyond traditional methodologies (direct observation). First, the ever increasing availability of movement data provides the opportunity to infer behaviour from movement types [5]. Second, behaviour can be remotely monitored through a variety of sensors [66,67]. The advantages of these two approaches with respect to direct observations are twofold: they limit the interference of the observer, and exponentially increase the range of analysis.

The challenge is **how to identify different types of behaviour from movement data**. Behaviour types are often extracted from trajectories with various forms of statistical modelling, including state-space models, various types of random walk models and behavioural change point analysis [68-71]. Alternatively, data mining techniques, such as clustering are used for this purpose [72,73]. Or movement-derived parameters, such as speed are used to classify behaviour types [74].

Another promising perspective is the simultaneous recording of movement and information derived from **other sensors**, especially accelerometers. Accelerometers measure changes in velocity over time in three dimensions at very high temporal resolutions (10 Hz). These data can be used to identify two types of patterns: first, it is possible to identify changes in body posture and behaviour and second, the variation in measurements has been linked to speed and energy expenditure [67,75,76].

When accelerometers are used in combination with a GPS tracking device, acceleration data can be used to segment bird trajectories into behaviour classes including flying, foraging, body care, standing and sitting [76,77]. Accelerometer data can also be linked to GPS and magnetometer data [78] or alternatively in combination with a gyroscope, which measures the orientation and aids the accelerometer in high-frequency motion situations [79].

In many of these cases, video observations are collected simultaneously with locational and/or accelerometer data. Video footage serves as ground truthing for behaviour types, automatically derived through data mining [76,78,79]. Behaviour can also be identified directly from video-tracked data [80] or movement parameters can be derived from 3-D trajectories derived from video using computer vision: an example is data mining of movement parameters on 3-D trajectories of zebrafish movement [81].

MOVE studies utilise methods from computational geometry, spatial data mining and visualisation/visual analytics to **support behaviour identification from movement data**, as described in the second part of this review.

### T4: Linking movement data with environmental context

The movement of an organism is affected by the internal state of the organism and by the external factors including environmental context of the individual’s location [1]. External factors affect the movement in many ways. They can trigger behavioural patterns or migratory decisions. Animals may decide to move at times with conditions supportive for a particular movement type while allowing them to optimise energy expenditure. Movement is therefore often linked to spatial and temporal variability in environmental conditions [82].

To investigate the influence of environment on movement, tracking data can be complemented by environmental data from many sources and of many types. Some studies incorporate remotely sensed satellite data with trajectories [82-85], others link trajectories to either meteorological information such as wind direction and speed [86] or to weather radar data [87,88]. For interested readers, there is an on-going COST Action ES1305 on this topic: “European Network for the Radar surveillance of Animal Movement (ENRAM)”, 2013–2017. For land animals, weather effects (snow) and topographic factors (slope) can be linked to movement [47]. Trajectories can be linked to field data on home range productivity and related indices derived from remotely sensed data, e.g. the normalised difference vegetation index (NDVI) [89]. For marine mammals (whales, dolphins, seals), passive and active acoustic monitoring is used in combination with trajectories [90].

An alternative to external environmental data is to include more than one sensor on an animal tag. This is particularly common for marine animals, where tags are traditionally referred to as bio-loggers and incorporate both locational and environmental sensors [5,8,66,91]. Frequently used are oceanographic sensors, such as the conductivity, temperature and depth (CTD) loggers and specific sensors for salinity, turbidity, fluorescence, level of chlorophyll, presence of cyanobacteria and other oceanographic parameters [92-94]. Locational sensors for marine species are often a combination of GPS tracking devices and various marine Satellite Relay Data Loggers (SRDL), which measure location, speed and depth of the diving animal [92,95]. Researchers have used data from combined oceanographic and locational sensors to model not only animal movement, but also the state of the oceans in remote areas inaccessible for human observers, but which animals (e.g. polar seals) visit regularly and periodically [93,94].

In many studies, movement data are linked to environmental or other sensor data through **trajectory annotation**. This is a process that semantically enriches trajectories with environmental and sensor information at each location and time [86,96]. Due to large data volumes of both tracking and environmental or sensor data, this has to be done automatically and systems are being developed to support this procedure. An example is the Environmental Data Automated Track Annotation System (EnvDATA) that allows annotation of trajectories from the animal movement online data repository Movebank (www.movebank.org, [10]) with satellite remotely sensed information [82]. Another example is the spatial database of environmental data linked to the species-distribution range in the Eurodeer project (www.eurodeer.org, [47]).

Is semantic trajectory annotation the best way to connect movement and environmental data? The problem is that these two types of data are collected at different spatial and temporal scales. Animal movement can be collected with 1 Hz resolution at times with accurate GPS locational measurements down to sub meter accuracy, while satellite data for a particular location may only be available from half-daily or daily satellite passes and collected at spatial scales of several tens or hundreds of meters [82]. Data pre-processing measures (e.g. spatio-temporal interpolation or aggregation) are therefore required prior to semantic trajectory annotation. This process may propagate the uncertainty related to coarser spatio-temporal resolution of environmental data into higher-resolution trajectory analysis. A question is therefore how to capture and describe or eliminate the uncertainty resulting from matching the spatially and/or temporally misaligned data. This is an area where MOVE contributed with **cross-scale analysis for context-aware trajectory analysis**.

## Review

This section presents methodologies for movement analysis and visualisation developed in MOVE grouped in the following categories:

1. Geometrical analysis of trajectories
2. Similarity and clustering
3. Visualisation and visual analytics

For each of these categories we describe methods developed in MOVE and link them to the ecological themes from the first part of this review.

### Category 1: Geometrical analysis of trajectories

Geometrical analysis of spatial data is developed in a number of information sciences in which methods are based on geometry and location of data entities in space. In terms of trajectories, geometrical methods are useful for a number of problems relating to the form and relative positions of trajectories in the 3-D (or 4-D) physical framework space of 2-D (or 3-D) position and time. In MOVE, geometrical analysis was represented by computational geometry [97] and GIScience [98] and the studies can be grouped into the following topics:

- trajectory segmentation,
- identifying a representative path from a set of trajectories,
- scale-dependent geometric analysis and
- identification of spatio-temporal patterns.

These methods may be useful for themes T2 and T3 (Table 1).

**Table 1:** **MOVE studies in Geometrical analysis, categorised per method type vs. ecological themes (T) they address**

	**Sources relevant for ecological themes**	
**1. Geometrical analysis**	**T2**	**T3**
Trajectory segmentation	104, 105	99-104
Identifying a representative path	106-110	
Scale-dependent geometric analysis	113	114, 115
Identification of spatio-temporal patterns	116, 117	

T2: Identification of spatio-temporal patterns in movement and T3: Classification or identification of behaviour from movement data.

### Trajectory segmentation

**Figure 1:**
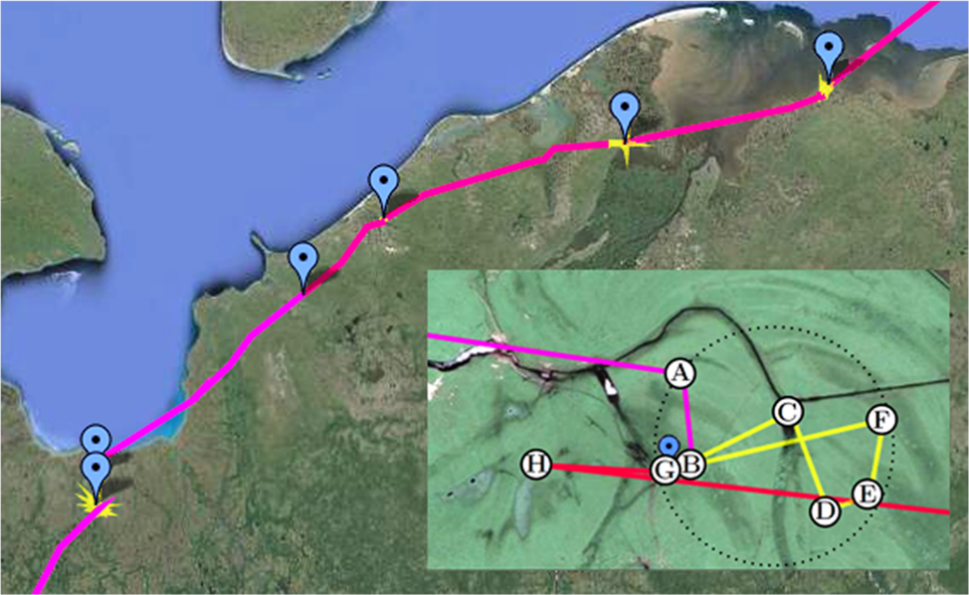
**(after** **[** 103 **]** **).** A geometric segmentation of a trajectory. Red/pink segments are migration flight, yellow segments are stopovers (one stopover is shown in more detail in the lower right corner). Blue markers indicate end of a stopover. Stopovers are described by staying within a bounded activity radius for at least 2 days. This is a conjunction of a monotone-decreasing criterion (if the sequence **(B, C,…, G)** stays in a disk of small radius then so does every subsequence, e.g. **(D, E, F)**) and a monotone-increasing criterion (if the sequence **(B,…, G)** corresponds to a duration of at least two days then so does every sequence which includes it, e.g., **(A, B,…,G, H)**).

Geometric trajectory segmentation refers to the problem of splitting a trajectory into pieces (referred to as segments) such that each piece fulfils a geometric criterion. If the geometric criteria characterise behaviours of a moving entity, this problem is closely linked to classification of behaviour (T3). Buchin et al. [99] developed a segmentation method for animal trajectories based on individual movement states of the moving object (animal). They look at different types of bird movement (flying, foraging and resting) and by linking these to different types of geometrical properties of trajectories (location, speed, angular range, heading, time, etc.) they developed a method that automatically segments bird trajectories into segments that correspond to these states (Figure 1). The results of such an algorithm can then be used for further exploratory ecological analysis of birds’ movement.

Geometric segmentations are often optimised based on a set of general spatio-temporal criteria. This means that the methods aim to minimise the number of segments while guaranteeing that each segment fulfils one of the criteria [100]. Various approaches are concerned with the scale at which the criteria are satisfied, for example, if they are satisfied on one particular segment and all its sub-segments or all larger segments that include this one particular segment. An efficient framework for geometric segmentation was proposed by [100] for criteria that are monotonically decreasing, i.e. if they are fulfilled on a segment, then they also are on its sub-segments. This framework was extended [101] to include combinations of monotonically decreasing and increasing criteria (as in Figure 1). If segments are not restricted to start at points, the general segmentation problem becomes computationally intractable [102], but can still be solved efficiently for monotonically decreasing criteria [100].

Geometric segmentation can also be linked to statistical analysis, which is an approach that may be more familiar to ecologists. Alewijnse et al. [103] propose a model-based approach to segmentation of movement data. In this approach, a segment is defined by a uniform model parameter and an information criterion is used to select the number of segments. This approach assumes little knowledge on geometric characteristics of the input trajectory data, yet it identifies the optimal segmentation by optimising the information criterion, linked to the complexity of the movement model. This particular approach uses Brownian bridges, but can be generalised to any parameterised movement model.

Segmentation methods were also developed in MOVE for other types of trajectories. Sester et al. [104] present a method to link segments to human movement behaviour. Their segmentation is based on identifying important places from trajectories (most frequently visited places for the longest time). Segments between these places are classified based on movement parameters linked to travel mode (walk, bus). This approach could serve to identify equivalent patterns in animal movement, for example, important stopover places of longest duration in data on annual migration (theme T2).

Panagiotakis et al. [105] present a method for segmenting trajectories into representative and non-representative segments based on other nearest trajectories. They use vehicle trajectories for their experiments. As their data vary according to spatial and temporal density in location sampling, this could be relevant to animal trajectories obtained with irregular sampling schemes.

### Identifying a representative path from a set of trajectories

Another frequent geometric problem is how to identify a representative path for a set of given similar trajectories. Is there an optimal route that can be used to represent this set? How can this route or path be defined, while the path may or may not be one of the actual trajectories? This could be useful in ecological terms for theme T2: identification of routines and consistency in migration [49,50].

**Figure 2:**
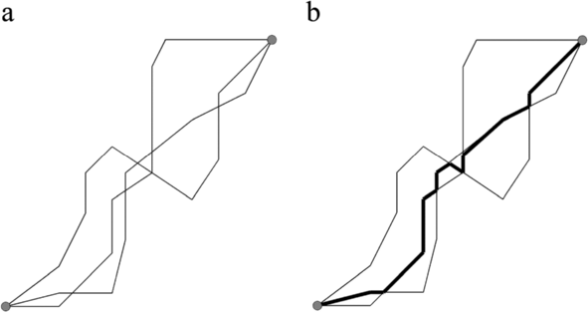
**(after [** 106 **]). a)** three trajectories with a common start and end point and **b)** a median trajectory (bold) representing these three trajectories. The median trajectory is built of segments of the original trajectories and switches the original underlying trajectory at each intersection.

In MOVE, Buchin et al. [106] introduced a computational geometry approach to compose such a representative route from parts of the actual trajectories. Here the trajectories do not need to be temporally correlated, but just need to follow a similar spatial route. They call their representative route a median trajectory and build it from pieces of the trajectories in the data set (Figure 2).

Identifying a representative route from a set of trajectories is a common topic in GIScience and there are many approaches outside MOVE. Brudson [107] uses principal curves to identify the most probable route from a set of GPS pedestrian trajectories. Similar methods are widely used in navigation and even developed for reconstruction of representative 3-D trajectories: [108] reconstruct 3-D bicycle tracks from GPS trajectories – a method that could be of interest for movement of animals freely moving in 3-D (birds, sea mammals). In MOVE, Etienne et al. [109] developed a method to identify the main naval route from a set of vessel trajectories, sampled at equal times. Pelekis et al. [110] take an alternative approach and consider the uncertainty in trajectory measurements by constructing a fuzzy vector representation of each trajectory. They use this representation to construct a so-called centroid trajectory as the representative path based on density of trajectory points at each moment in time.

Note that purely geometrical methods of identifying a representative trajectory have certain limitations when considering animal data. For example, a median trajectory provides a population-average summary of paths, however, its characteristics may not match up with the movement of any particular individual. The difference between the average model of movement vs. individual models is a well-known issue in ecology [111], which is analogous to the problem of global vs. local modelling in spatial statistics. We discuss this similarity in concepts in ecology and GIScience as part of one of the future challenges.

### Scale-dependent geometric analysis

Movement characteristics are influenced by processes operating at different spatio-temporal scales [1]. There is therefore a need to support movement analysis across different scales and investigate how these multiple-scale processes act together. On this topic, movement ecology could benefit from the dependency of geographic phenomena on spatio-temporal scale, which is one of the most well-known and longest-standing topics in GIScience [112].

In MOVE, a number of GIScience studies explored the issue of scale. Laube and Purves [113] investigate how temporal scale affects the calculation of movement parameters (speed, sinuosity and turning angle) of animal trajectories. They demonstrate what they call the “granularity grief”: the fact that derivation of any kind of movement parameter from trajectory data is influenced by the temporal sampling rate and thus scale-dependent. They further demonstrate the relationship between uncertainty in individual GPS measurements at different scales and how these affect the fine-scale movement descriptions. This could be of interest to theme T2.

In a recent study on cross-scale movement analysis [114] show how the derivation of different movement parameters over a range of spatial and temporal scales significantly improves the subsequent classification of movement behaviour from a set of zebrafish trajectories, compared to a single-scale approach. This could be relevant to theme T3. Soleymani et al. [115] use cross-scale extraction of movement parameters and context information as input features to detect foraging behaviour in GPS trajectories of wading birds. Their results suggest that it is possible to classify, with high accuracy, fine-grained behaviours based on high-resolution GPS data, providing an opportunity to build a prediction model in cases where no additional sensor (e.g. accelerometer) or observational data is available.

### Identification of spatio-temporal patterns

This sub-topic is linked to theme T2. Predating MOVE, a study [116] provides a general framework of spatio-temporal movement patterns that can be identified from trajectories. In MOVE, Orellana et al. [117] propose a method to identify suspension patterns in movement, which represent an attraction or an obstruction for the moving object.

### Category 2: Similarity and clustering

A frequent task in trajectory analysis is to partition the data into groups of similar trajectories. In data mining, clustering takes a set of data objects and partitions these into groups (clusters) so that the objects in the same group are more similar to each other than to objects in other groups [118]. The procedure consists of two steps: first, a similarity measure has to be defined based on the data domain and second, a grouping procedure is used to partition data into clusters based on similarity between data objects. In this section we focus on **similarity measures for trajectories** – for reviews of clustering methods see [118,119].

We group trajectory similarity methods from MOVE based on what part of trajectory data space they consider. Trajectories are spatio-temporal data and their data space can therefore be partitioned into three separate sub-spaces: spatial part (location), temporal part (time) and attribute part (derived movement parameters or other). The following trajectory similarity measures were developed in MOVE:

- geometrical similarity, based on location and time only,
- similarity based on physical attributes of movement (speed, acceleration, direction, etc.) and
- context-aware similarity based on a combination of attributes.

For ecological themes, similarity and clustering of trajectories can support identification of particular spatio-temporal patterns in movement, that may also be related to behaviour, thus supporting themes T2 and T3 (Table 2).

**Table 2:** **MOVE studies in Similarity and clustering, categorised per method type vs. ecological themes (T) they address**

	**Sources relevant for ecological themes**		
**2. Similarity and clustering**	**T2**	**T3**	**T4**
Geometrical grouping and similarity	121-128		
Similarity based on physical attributes of movement	123, 129–132, 135, 136	114, 130, 133, 134	
Context-aware similarity			137, 138

T2: Identification of spatio-temporal patterns in movement, T3: Classification or identification of behaviour from movement data and T4: Linking movement data with environmental context.

### Geometrical grouping and similarity

Geometrical similarity methods rely on the notion of distance between trajectory points, which is usually a spatial distance. Originally such measures were developed to compare shapes of polygonal lines and only considered locations of points. Traditional geometrical similarity measures include Euclidean distance between each pair of points, Hausdorff distance that identifies the largest distance from a point on one trajectory to the closest point on the other trajectory, and Fréchet distance that takes into account the location and ordering of trajectory points and is sometimes also called the dog-walking distance (it represents the minimum length of a leash between two objects, i.e. a person and its dog, that move along respective trajectories without backtracking). More recently the temporal aspects of movement are also considered [120].

In MOVE, Buchin et al. [121] compare data points at equal times, whereas [122] compare data points with a bounded local time shift. Pelekis et al. [123] develop a similarity measure based on the area between two trajectories. Merki and Laube [124] define a set of grouping patterns (pursuit, escape, avoidance and confrontation) and present algorithms for their detection. Outside of MOVE, Rinzivillo et al. [125] use several geometric similarities in their clustering. First they consider only the distance between the start and end points of each trajectory; then they use distances between a selected number of sampled points on each trajectory and finally the smallest distance between two trajectories at a certain time.

**Figure 3:**
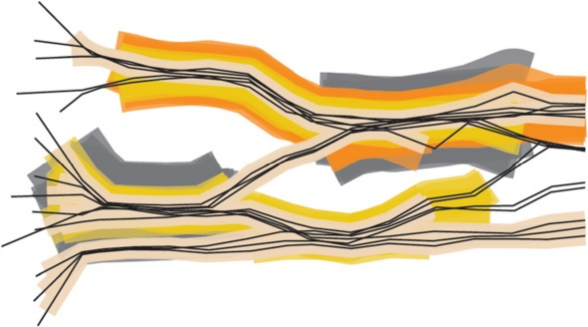
**(after [** 127 **]).** Progression of a set of trajectories through time (represented as horizontal axis and progression is from left to right). Colours indicate groupings of various sizes based on location and proximity of moving objects at that moment. A beige group has three objects, a yellow one four, an orange one five, a grey one six. At each moment in time the grouping is maximal.

Several of these methods have been used for clustering of sub-trajectories to identify entities moving in a group over some period of time [120,126]. Groups occur when a large set of moving entities moves sufficiently close for a sufficiently long time and may split or merge with other groups. This may be useful for identification of dynamic interaction patterns (T2). In MOVE, Buchin et al. [127] propose a representation of how such groups evolve over time (Figure 3). Outside of and pre-dating MOVE, methods that identify similar sub-trajectories are often based on either dynamic time warping or identification of the longest common subsequence [128].

### Similarity based on physical attributes of movement

A number of recent studies in GIScience approaches trajectory similarity by looking at physical properties of movement, which include physical descriptors of movement (speed, direction, acceleration, turning angle, angular speed) and path shape properties (curvature, sinuosity, tortuosity). These quantities are either measured by the tracking device or derived from trajectories and are referred to as **movement parameters** [116,129]. In terms of movement ecology, clustering based on these parameters can be used for inference about movement behaviour [130] and thus contribute to theme T3. In the following we describe MOVE studies that use movement parameters to define trajectory similarity.

Pelekis et al. [123] introduce four types of trajectory similarity, out of which two are geometrical (spatio-temporal similarity and spatial-only similarity) and two based on movement parameters (speed/acceleration-based similarity and directional similarity).

Dodge et al. [129] define the term “movement parameter” and use various individual parameters (velocity, acceleration, turning angle, displacement, straightness index) to build temporal movement parameter profiles. These profiles are used to decompose trajectories into segments of homogeneous movement. Dodge et al. [131] use the conceptual space of movement parameters (MP space) to compare two or more trajectories and define their similarity based on the temporal progression of the respective trajectories in the MP space. They use speed, azimuth, turning angle and acceleration to identify groups of concurrent and coincident trajectories. Concurrence is defined as similar progression through MP space and coincidence as similar progression through 3-D space, a space-time cube (STC, see visualisation section). The study is performed on a well-known trajectory data set of hurricanes in the Atlantic. Dodge et al. [132] extend their 2009 segmentation method with an alternative similarity measure based on producing a string of symbols for each temporal profile in the MP space and using a modified string edit distance metric for trajectory clustering. Soleymani et al. [114] use the MP space parameters for cross-scale spatio-temporal identification of different animal behaviours.

McArdle et al. [133,134] combine physical properties of movement in the space-time cube with time series clustering methods to classify a set of pedestrian tracking trajectories into several behaviour types. They decompose the 3-D space-time cube into two 2-D projections of time vs. one of the two geographical coordinate axes and then compare similarities of trajectories in each (or both) of these projected spaces based on their shape as mathematical curves.

**Figure 4:**
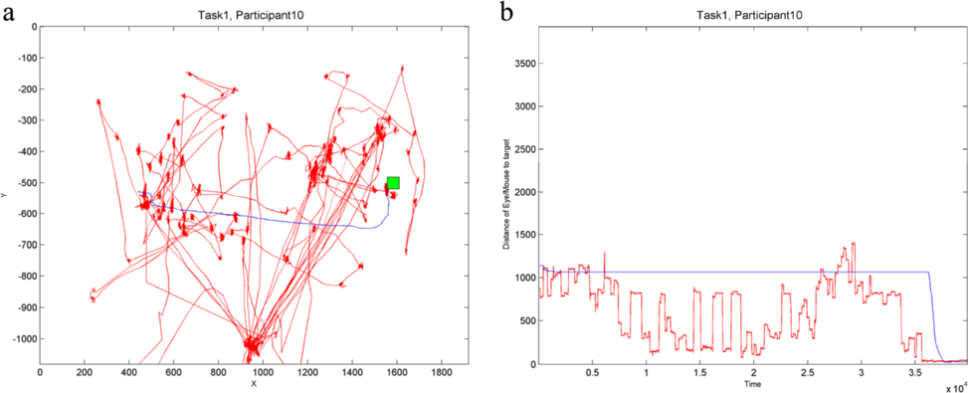
**(after [** 135 **]).** Eye and mouse trajectories in a visual search task: the participant was asked to identify the target (green square) on a map (not shown) and click on it. **a)** Eye and mouse trajectories generated in this task. **b)** Time series plot of distances from eye & mouse to target vs. time. In both charts, eye is in red, mouse is in blue.

Çöltekin et al. [135] use a set of eye tracking specific movement parameters for eye- and mouse-tracking trajectories obtained during a task of visual search on a computer display to investigate hand-eye interaction. In Human-Computer Interaction (HCI), eye tracking is a way to evaluate the usability of visual interfaces, where trajectories of gaze on the screen are collected using an eye tracking device. Trajectories of mouse movement on the screen are also collected for a similar purpose and this study investigates if there is a connection between the two trajectory types using trajectory analysis. Movement parameters used to evaluate similarity of the eye and mouse movement are distances from gaze or mouse to target and distance between gaze and mouse trajectory (Figure 4). Such studies, while using trajectory data from an unrelated domain (HCI), could be relevant for analysis of dynamic interaction patterns in theme T2, as data type (trajectories) and conceptual formulation of the problem (interaction of two moving objects, in this case gaze and mouse pointer on the screen) are the same as in the dynamic interaction problem.

Outside of MOVE, Ranacher and Tzavella [136] provide a broader review of physical movement trajectory similarity measures in GIScience.

### Context-aware similarity based on a combination of attributes

As discussed in theme T4, animal movement is inherently embedded in the environmental context. In MOVE, several developments integrated contextual information into similarity analysis contributing to theme T4.

Buchin et al. [137] present a method for integrating land cover information into similarity analysis. They extend geometric similarity (equal time distance, Hausdorff distance and Fréchet distance) with context distance, which for their hurricane trajectories consists of external and internal factors that influence hurricane movement. External factors include atmospheric conditions (temperature, air pressure), land use (land, sea) and topography of the region, while internal factors relate to properties of hurricanes themselves (intensification, wind speed, move speed, diameter). Their method is able to distinguish between hurricanes that have a similar spatio-temporal track, but different context.

**Figure 5:**
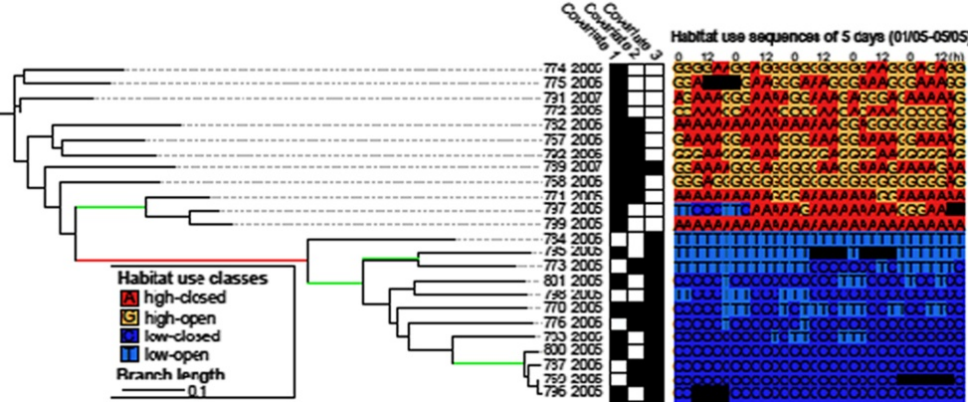
**(after [** 138 **]).** Tree classifying individuals based on spatio-temporal sequential habitat use during May-June. An extract of the habitat use sequences for the first five days (01/05 – 05/05) is shown. Covariates can be associated to each individual and help to identify relations between identified clusters of similar sequential habitat use and animal characteristics.

In a more sophisticated attempt at incorporating context into similarity analysis [138] consider the temporal variation in the sequential use of environmental features (relevant to T2 and T4). Their objective is to explore spatio-temporal patterns in the sequential habitat use by animals and they propose a tree-based approach using sequence alignment method (SAM) [139]. Sequences are constructed from roe deer trajectories by linking location to four habitat use classes, defined from two geographical parameters: habitat type and elevation. SAM is used to cluster the sequences into dendrograms (Figure 5), where clusters of similarly-moving animals can be identified at different levels of detail. By linking additional covariates to their results, they explore the relationship between identified clusters and animal characteristics.

### Category 3: Visualisation and visual analytics

Two final MOVE disciplines interested in movement are visualisation and visual analytics [140]. Vision is the most important sense in communication between humans and computers and visualisation plays an important part in cognitive processing. It supports data analysis in several ways: it provides an ability to portray and understand large amounts of data; it allows identification of patterns in the data that were not previously evident and thus supports hypothesis generation; the patterns are identifiable at large and small scales; and problems with the data can become quickly apparent [140]. Different visualisation communities include scientific visualisation, information visualisation and visual analytics. The first two portray spatial and non-spatial data respectively [140,141], while visual analytics combines human reasoning and pattern recognition ability with the computational capabilities of a computer to support a more efficient data analysis [142-144].

The increased recent availability of all types of movement data has kick-started visualisation and visual analytics developments for movement, resulting in a wide variety of methods and tools [145-147]. Many of these applications, while firmly anchored in visualisation or visual analytics, use animal movement data as inspiration [148-150] as did many of the participants in MOVE. We categorise MOVE contributions into the following three categories:

- Spatio-temporal visualisations: space-time cube and other approaches
- Visual aggregations: geometric aggregations and kernel densities
- Visual analytics of movement

Many of these studies (Table 3) contribute to themes T2 and T4 by providing the ability to visually identify various previously unknown spatio-temporal patterns. Aggregations support theme T1 by visualising temporal dynamics of space use.

**Table 3:** **MOVE studies in Visualisation and visual analytics, categorised per method type vs. ecological themes (T) they address**

	**Sources relevant for ecological themes**		
**3. Visualisation and visual analytics**	**T1**	**T2**	**T4**
Spatio-temporal visualisations		133-135, 157, 158, 160, 161, 164-167	
Visual aggregations	15, 62, 175-190	145, 168–173, 182, 188	
Visual analytics, attribute visualisations, linked views		135, 146, 147, 158, 165, 166, 170, 173, 183, 193-198	193, 194

T1: Spatio-temporal dynamics of home ranges and utilisation distribution, T2: Identification of spatio-temporal patterns in movement and T4: Linking movement data with environmental context.

### Spatio-temporal visualisations: Space-time cube and other approaches

The problem of representing time along with two spatial dimensions has a long tradition in GIScience [151,152]. In 1970s, a branch of geography called time geography established one of the most frequently used visual representations of interlinked geography and time: the space-time cube (STC) [153]. In an STC, the spatio-temporal data are shown in a 3-D space, where the bottom 2-D plane represents the 2-D geographic space and the third axis represents time. The main assumption of time geography is that geographic space and time are inseparable and the STC was developed to portray this assumption in a visual manner. Since then, the STC has become popular in GIScience for visualisation of human activity patterns [154-156]. In the visualisation community (and outside of GIScience), the STC popularity to show the temporal component of any type of spatio-temporal data (not just trajectories) has also recently increased [157].

In MOVE, the basic form of STC for trajectories (where trajectories are shown as polylines in the STC space) was used in several studies. McArdle et al. [133,134] superimposes the STC on a virtual globe (Google Earth), so that the third dimension consists of a sum of elevation and time, thus making it appropriate for locations with flat terrain. Of note is the linkage to Google Street View [134], which allows for visual ground-truthing of automatically-derived stopping points (e.g. one frequent stopping point turned out to be a shop, another a dentist’s office). This may be of interest to ecologists who try to understand stops in migration (relevant to T2). At present the availability of Street View limits this kind of exploration to specific countries, however, the coverage is likely to be extended in the future.

Çöltekin et al. [135,158] apply the STC concept to visualise interaction between gaze and mouse trajectories. Here the base 2-D plane represents the display of the stimulus on the computer screen and the third dimension represents time. Their approaches might be of interest to ecologists who are exploring the dynamic interaction between animals (relevant to T2), to which the interaction between the eye and mouse movement is analogous.

The inherent inseparability of space and time in the 3-D STC is difficult to achieve if only spatial or only temporal visualisations are used. However, as a 3-D display, it is complex to use and its usability needs to be empirically examined [159]. MOVE contributed to this through usability experiments in which the authors deconstructed the STC from the traditional cartographic point of view and made recommendations about the strengths and weaknesses of this popular 3-D display [160,161].

STC is only one of many temporal visualisations (see [162,163] for reviews). In MOVE, several collaborations between practitioners and visual developers resulted in alternative temporal displays, some specifically aimed at animal ecology. One study [164] developed bespoke spatio-temporal displays of bird migration patterns (relevant to T2). They focus on a specific set of migration-related questions, such as timing of annual migration, route fidelity and identification of stops. They also explore how these events relate to the time of the year (onset of spring) and how the spatio-temporal patterns vary between individuals and years. Another collaboration between visualisation experts and ecologists developed space-time visualisations to explore changes in biodiversity [165], using timeline and species density displays, relevant to visualising temporal dynamics of population distribution (T2).

In MOVE, Zhang et al. [166] present a timeline display developed for a set of identification, localisation and movement comparison tasks to study urban movement trajectories. Outside MOVE, Wang and Yuan [167] use a similar set of temporal visualisations, including a timeline, a straightness plot and others to investigate spatio-temporal patterns in urban movement.

### Visual aggregations: geometric aggregations and kernel densities

When movement data sets are large, visual displays that show all trajectories become unsatisfactory, as the overprinting and clutter increase to the point that no patterns can be reliability identified anymore. In MOVE, Netzel et al. [168] have investigated how different line rendering styles can help improve the perception of dense visualisations of trajectories and do so using oystercatcher trajectories as a case study.

Even with improved trajectory rendering methods, the overplotting problem eventually becomes too severe for complex and large data sets. In such cases a frequently used concept is visual data aggregation. Aggregation refers to combining several data elements into a single unit that is then shown in some other way than the original data would be. This operation reduces the size of the data to be displayed, while at the same time there is inevitable information loss as patterns are generalised. Andrienko et al. [145] present an overview of aggregation methods for movement data and [157] discuss this concept in the context of an STC. Studies in MOVE developed two types of aggregations: geometric aggregations (relevant for T2) and aggregations using kernel density estimation (relevant for T1 and T2).

#### Geometric aggregations

A common approach to aggregate movement data for spatial visualisation is edge bundling, which merges nearby sub-paths into one. It is often used for origin–destination data (movement data where only start and end points are known). Methods for edge bundling are popular and include force-directed approaches [169,170], combinatorial techniques [171] and image-based methods [172]. In MOVE, Hurter et al. [172] demonstrate the usefulness of edge bundling for simplifying and aggregating various types of movement trajectories, in particular eye tracking data. Höferlin et al. [170] enrich the aggregation of groups of trajectories by applying abstracted schematic rendering that reflects the aggregation process. Another MOVE study [173] incorporates the edge-bundling principle into a time lens display of car movement.

#### Aggregations with kernel densities

**Figure 6:**
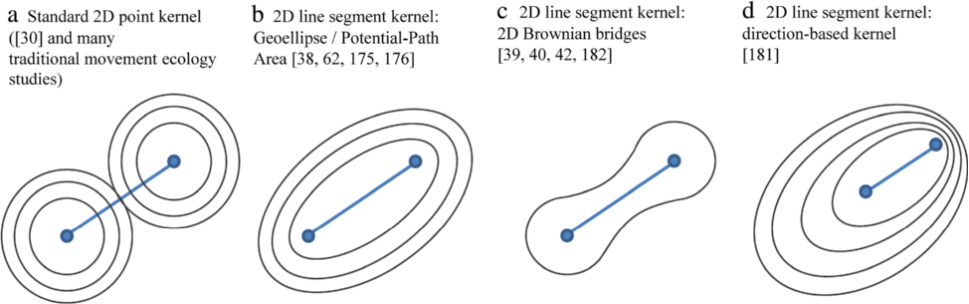
**(after [** 190 **]).** Two-dimensional kernels for trajectories that produce two-dimensional density surfaces. The point-based kernels in panel **a)** do not consider the temporal dimension of trajectory points, but treat them as independent observations in a point data set. Panels **b)**, **c)** and **d)** show line-segment kernels, where sequentiality of two consecutive trajectory points is taken into account in kernel construction.

As mentioned, the concepts of utilisation distribution and home range are often shown using kernel density estimators of trajectory points. These methods traditionally assume that the points form an independent sample taken from a static 2-D probability distribution of the individual’s locations and ignore the sequentiality of points. This is often addressed through sequential kernels where kernels are placed not over trajectory points but over trajectory segments and are added into a two-dimensional probability surface, as is normally done with point kernels. Note that for visualisation purposes we call a segment a line between two consecutive trajectory points. This is different from the segments in trajectory segmentation in geometric analysis, where a segment refers to a sub-trajectory, which may consist of any number of consecutive points. Figure 6 presents an overview of segment kernel approaches and lists relevant GIScience and ecological references, some from MOVE and some preceding MOVE.

In GIScience, segment kernels are often defined based on principles of time geography [153] and space-time probability prisms [174]. Considering movement in terms of space-time prisms results in elliptical kernels that define the area covered by all possible movement paths between two trajectory points. This is a popular GIScience approach to model animal movement [15,62,175,176]. In MOVE, researchers modelled vessel line density based on similar principles and convolution of density fields around two consecutive points [177,178]. Vessel line densities were linked into interactive systems with other geovisualisations [179,180]. Other researchers in MOVE took into account acceleration and velocity of movement through directional segment kernels [181].

**Figure 7:**
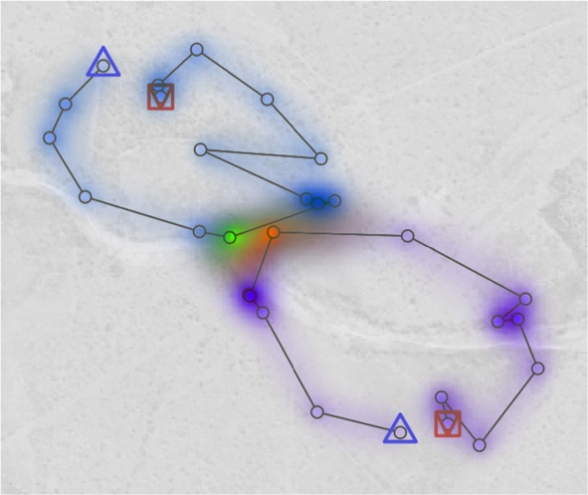
**(after [** 182 **]).** Two trajectories (blue/purple) with a potential encounter (red/green) computed based on the Brownian bridge movement model.

A MOVE collaboration addressed the problem of low sampling rate between consecutive trajectory points using Brownian bridges [182]. If the temporal sampling rate is too low for the linear movement between two observed locations to make sense, a segment kernel that takes into account movement uncertainty is more appropriate to use. Buchin et al. [182] develop their approach to visualise uncertainty in movement as well as identify interaction patterns such as encounter, avoidance/attraction, regular visits, and following (Figure 7).

**Figure 8:**
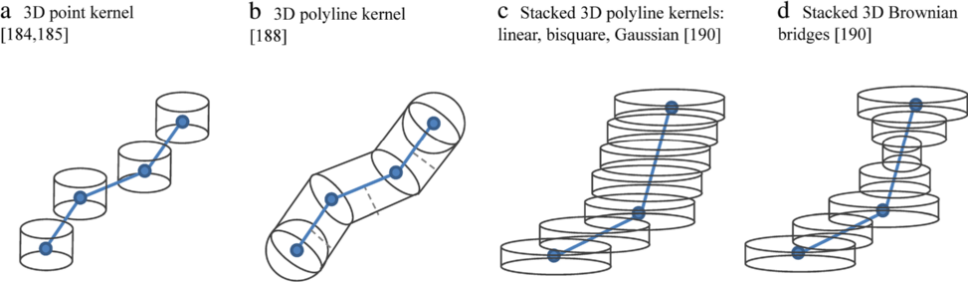
**(after [** 190 **]).** Three-dimensional kernels for trajectories that produce volumes in geo-time space. Panel a shows **a)** point space-time density with cylindrical kernels that do not take into account the temporal sequence of points in a trajectory. Panels **b), c)** and **d)** show polyline kernels, where there is one kernel for the entire trajectory (and not a separate kernel for each line segment). Distance from each voxel to trajectory in panel **b)** (shown in kernel with a dashed grey line) is calculated in 3-D, perpendicularly to the trajectory. Distance from each voxel to trajectory in panels **c)**-**d)** is measured at a constant moment in time (i.e. horizontally) and is calculated as 2-D distance. Panel **d)** shows the Brownian bridges version of the stacked 3-D kernel, where the width of the kernel at each moment in time depends on the position on the trajectory between each two consecutive points.

All of the kernel density models of utilisation distribution described so far, whether they incorporate time or sequentiality into the algorithm or not, are represented as surfaces in two geographical dimensions. MOVE researchers have combined the STC principle with a generalisation of the 2-D kernel density into three dimensions (Figure 8). A MOVE study used density of gaze points from eye tracking to enrich the STC visualisation of gaze points from a large number of eye tracking experiments by colour coding [183]. Outside of MOVE, a 3-D point kernel density in STC has been used in crime visualisation [184] and in spatial epidemiology [185]. Another GIScience study combined the 3-D point kernel density with the principle of space-time prisms in time geography to generate probabilistic space-time prisms for understanding the movements and activities of animals at fine temporal and spatial scales [186]. Also outside of MOVE, a recent ecological study [187] develops 3-D densities in real physical 3-D space (i.e. not the STC) taking into consideration elevation as well as the two geographical coordinates of location.

**Figure 9:**
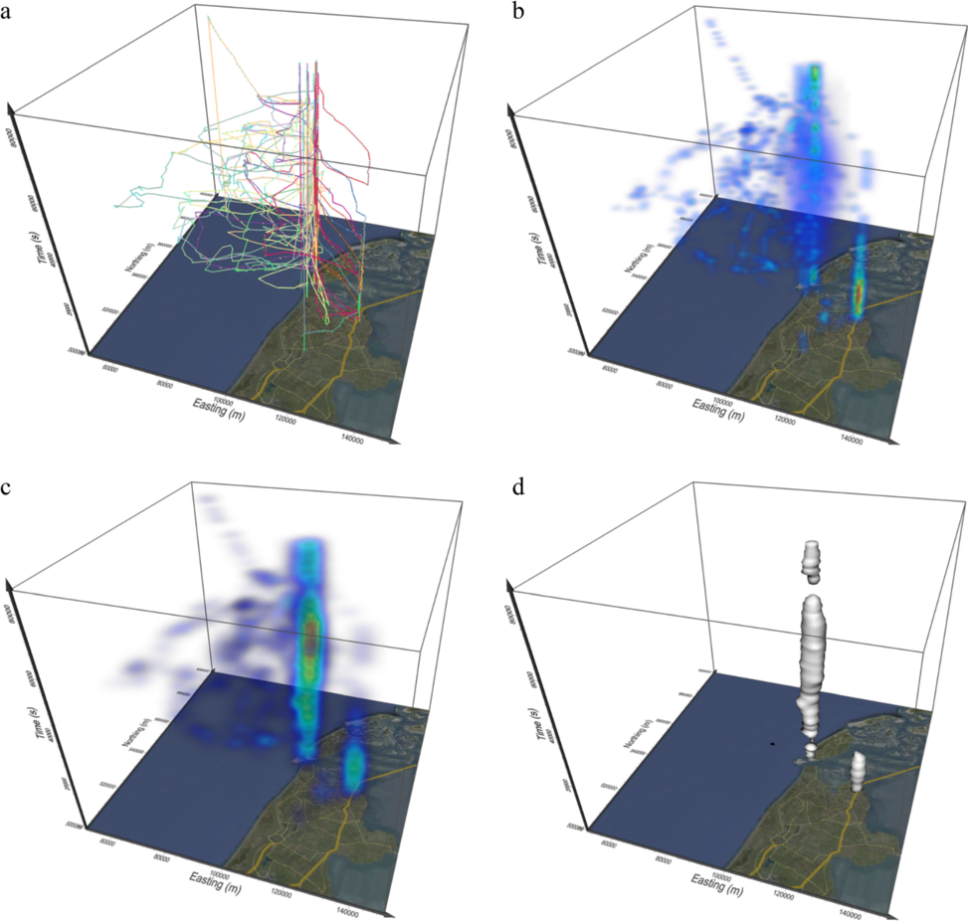
**(after [** 190 **].** Stacked space-time density of animal trajectories. **a)** Space-time cube representation of one month of trajectories of one individual bird. The x-y plane represents geographic space and the z-axis is time (0-24 hrs). **b)** Brownian stacked space-time density of the trajectories from the space-time cube. **c)** Gaussian stacked space-time density of the same data and **d)** isosurface of the highest values in the Gaussian density, indicating a temporal column and a space-time hotspot.

Generalising the concept of point density into a model that considers sequentiality of movement, one MOVE study developed the space-time density of polylines [188]. Here, the kernels are not calculated for each point nor each segment, but span the entire trajectory, which is represented as a polyline in the STC. The kernel around each trajectory is built within a volumetric union of rounded-cylinders, one cylinder for each trajectory segment. A further MOVE collaboration produced a faster optimised version of the 3-D polyline density, the stacked space-time density, and linked it to the concept of home range dynamics. This study also provides several alternative kernels, including a three-dimensional Brownian bridge kernel [189,190]. Figure 9 shows an example of the stacked space-time density for a month of daily trajectories of one lesser black-backed gull [190]. As volumes, such 3-D densities can be displayed in various ways, either using direct volume rendering or isosurfaces. They allow for identification of spatio-temporal patterns that are otherwise undistinguishable from spatial-only patterns in a standard 2-D density surface.

An HCI study [158] investigates the level of interaction between the eye and mouse trajectories using the 3-D stacked space-time density [190] combined with 3-D volumetric change detection methods to quantify the level of interaction between eye and mouse trajectories. In terms of movement ecology, this may be relevant to dynamic interaction (T2).

### Visual analytics of movement

A common design approach for visual analytics systems for highly-dimensional complex data is to use linked views, that is a set of interactively connected visualisations, each of which provides a different perspective on the data [191]. This methodology takes a set of data displays (each showing a selection of the given dimensions in some particular way) and then allows the interaction in one view (e.g. selection, zoom in, zoom out, etc.) to simultaneously modify displays in all views [192]. This is relevant to visual exploration of spatio-temporal data, since it enables generating a unique spatial, temporal or spatio-temporal perspective on a pattern appearing in a linked attribute-only view [144].

Many MOVE studies mentioned above employ the linked views paradigm (e.g. [165,166,183]). Others include not only spatio-temporal displays, but also attribute visualisations. Tominski et al. [173] introduce a trajectory wall. Here, car trajectories are represented as ribbons in a 3-D space, where the bottom 2-D plane represents the geographic space and the third axis the vehicle count. Ribbons are stacked over their geographic path and their segments coloured according to an attribute (speed). This view is interactively linked to a time lens, where clusters of temporally similar trajectories are shown using edge-bundling. The system allows identification of temporal patterns in car movement and in particular anomalies in regular flow, such as traffic jams.

Andrienko et al. [193,194] present linked views for a comprehensive visual exploration of any type of movement trajectories. These systems include STCs, a number of attribute visualisations, density maps, temporal visualisations and a number of other displays that allow incorporation of contextual information.

**Figure 10:**
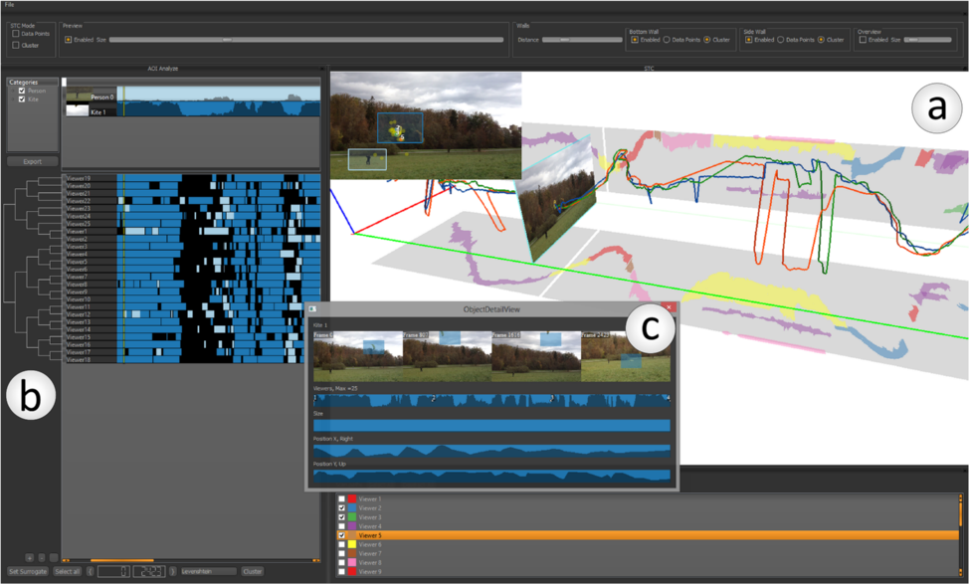
**Eye tracking data shown in the visual analytics tool ISeeCube [** 183 **,** 197 **] with multiple linked views.** The data shows gaze information from multiple participants watching the same video [189]. The visual workspace is separated into several regions that can be freely adjusted by the user. Region a) displays the scanpaths of selected participants in an STC (coloured lines), along with clustered gaze point data (coloured regions on the two grey walls). The STC also contains a snapshot of the video at a time frame that can be adjusted by the user. At the top-left of region **a)**, the same video replay is shown with two areas of interest marked by blue boxes (person and kite). Region **b)** provides a hierarchical clustering of the trajectories according to the similarity of their distribution of attention to areas of interest. Region **c)** shows the detailed information of one of the areas of interest (kite), including overall distribution of attention, as well as size and position of the area over time.

Another trajectory type widely represented in MOVE are eye movement trajectories, generated in HCI studies of visual displays. A number of MOVE studies used visual analytics for exploration of eye trajectories from such experiments. Andrienko et al. [195] introduce a comprehensive visual analytics methodology for exploring eye movement – their system is based on their previous work and visualisations [144,145,193]. Ooms et al. [196] use a combination of selection, simplification and aggregation operations to visualise and analyse patterns in eye tracking data. Kurzhals and Weiskopf [183] and [197] use a system with multiple linked views including a density-based STC representation for a set of gaze trajectories collected in an experiment with dynamic visual stimuli (videos, Figure 10). Finally, as described above, [158] and [135] use the STC and stacked space-time densities for concurrent visualisation of eye and mouse trajectories in an attempt to quantify the interaction between the eye and mouse.

The visual interfaces of visual analytics systems often include the aforementioned multiple linked views to provide a comprehensive visual representation of complex data. Another component that makes visual analytics particularly interesting for complex data is its incorporation of (semi)-automatic analysis methods. Here we can make direct use of the new MOVE trajectory analysis methods reviewed earlier in this paper. In particular similarity measures and clustering methods are useful because they allow us to group, aggregate and simplify large data sets. One example of MOVE research on this topic is a study that employs trajectory clustering to group the trajectories and then allow for interactive selection of subgroups and re-clustering [170]. The process allows for top-down exploration of the data set, repeatedly selecting one or a few clusters and re-clustering remaining trajectories. Clustering supports faceted exploration that allows to cluster trajectories according to a variety of similarity measures between trajectories (e.g. coverage, distance between means, distance between standard deviations) and facets (related to geometric information from the trajectories, e.g. position, velocity, direction of motion, time, and object class). Another example from MOVE is the analysis of gaze trajectories with the system by [183] and [197,198]. They support spatio-temporal clustering of gaze points on trajectories, as well as hierarchical clustering of the sequences of gaze trajectories based on some distance metric. Many of these metrics are based on string metrics such as the Levenshtein distance [199], which measures the difference between two words (sequences of characters) based on how many single-character edits (insertions, deletions, substitutions) are needed to convert one sequence into another. In [197] the Levenshtein distance is used to compare and cluster gaze trajectories represented as a string of subsequently viewed areas of interest.

A comprehensive overview of spatio-temporal visual analytics for movement is given by [146,147].

## Conclusions

Interdisciplinary collaborations such as the ones fostered in MOVE and described in this paper are reducing boundaries between disciplines that are interested in movement. To conclude, we propose as set of challenges that will be important to address in continued interdisciplinary collaborations between animal ecologists and ICT researchers. We identified five challenges, three based on specific themes in movement research and two more general ones, linked to characteristics of disparate scientific communities interested in movement.

### Challenge 1: Navigation

An important topic in animal movement analysis is the question of navigation, specifically long-distance animal navigation [48]. How do animals navigate in their migration? Some species (terrestrial birds) may exhibit genetically or culturally inherited patterns, others (pelagic birds) do not, and other as yet unexplained mechanisms seem to account for their migratory navigation control [50]. Yet other species may rely on map related cues and relationships between celestial and magnetic compass for their orientation [48]. Long-term tracking can assist investigations into navigation and migration mechanisms, but this has to be combined with behavioural experiments and exploration of internal mechanisms, such as sensory perception, neurobiological state and genetic characteristics of migrant animals. Identifying spatial principles of long-distance animal navigation from such a complex set of sources will require interdisciplinary collaborations of the type that MOVE has shown, but on a broader scale with inclusion of biology, genetics and neuroscience. It will also require simulation methods to efficiently generate null model trajectories for long-haul displacements [200].

### Challenge 2: Spatio-temporal dependency and heterogeneity

Movement trajectories are a special type of spatio-temporal data, that is, data with specific geographic and temporal location. Since recorded positions in a trajectory are not random, but are generated by continuous movement, the points in a trajectory are highly correlated in both space and time [5,201]. Indeed, the higher the temporal frequency of collection, the higher the correlation. This property is called the **spatio-temporal dependency** or the spatio-temporal autocorrelation and is a well-known issue in GIScience and spatial statistics [98,202-204]. A recent movement ecology study [205] incorporates geostatistical semivariogram modelling into movement analysis and another recent GIScience study incorporated spatial statistics measures (Getis-Ord Gi* statistic) with kernel density estimation and spatial data mining to identify periodicity patterns in migration trajectories of Arctic Barnacle Goose [206]. However, these are just first attempts and movement ecology could benefit from other developments in spatial and spatio-temporal statistics.

The second well-known property of spatio-temporal data is **spatio-temporal heterogeneity** or the property of geographical processes to vary over space and time [98]. Global statistical models used on spatial data often average characteristics from each location into descriptors that are valid over the entire area, but in fact are not valid anywhere. To address this, spatial statistics uses various local models, such as geographically and temporally weighted methods [203,204] to disaggregate descriptions of processes to individual locations in space and/or time. In ecology, an analogous problem is the distinction between modelling of movement at the population and individual levels [111] and exploration of local modelling could perhaps help address this problem in alternative ways.

### Challenge 3: Human movement behaviour vs. animal movement behaviour

Recently, a number of studies in computer science focused on identification of human movement behaviour patterns from GPS trajectories. These studies look at identification of significant places (i.e. locations which play an important role in the activities of a user) [207-209], classification of human behaviour in these places [210,211] and analysis of spatial interactions between significant places identified from trajectories [212]. In addition to this, new tracking technologies for observation of human movement have been deployed as alternatives to GPS technology: an example are short range wireless technologies such as Bluetooth [213-216] and wireless networks [217].

The deployment of wireless sensors is a relatively new avenue in biologging sciences, with an ever increasing number of wildlife studies using them as ‘proximity sensors’ [218-221]. An interesting problem is therefore the question if and how much are human-centric movement methods and technologies transferrable into the animal-tracking context. For example, Bluetooth experiments generate a data set of flows between sensor locations; the animal-tracking analogue could be flows between most-visited places in a home range, which can be detected by deployment of wireless sensors on animals and in the environment [219]. Alternatively, methods for human significant places could be used for identification of animal significant places and the movement between these further investigated with human-behaviour related methods, such as spatial interaction.

### Challenge 4: Statistics vs. exploratory data analysis

Ecology as a field is geared towards a hypothesis-driven approach that seeks confirmation through statistical testing. Indeed, most methodological questions on the recent list of one hundred fundamental ecological questions [2] are of a statistical nature. The information sciences are at the other side of the spectrum, geared towards exploratory data analysis, data-driven methods, data mining, visualisation and visual analytics. These perspectives are complementary and can enhance each other, but the challenge is how to best communicate and inform and learn from each other in achieving the goal: developing new methodologies for the analysis of large and complex new ecological movement data. One of the areas that could contribute to this challenge is visual analytics, where the links between exploration/visualisation and statistical methods are already being developed [222,223].

### Challenge 5: Publication and dissemination

Interdisciplinary collaborations face the challenge of disciplinary differences in how results are published and disseminated in each discipline, as is well-illustrated by the vast variety of the publication venues in our literature list. In MOVE, animal ecologists paired up with researchers from GIScience and computer science, such as specialists in computational geometry, data mining, spatio-temporal databases, or visualisation. These disciplines and subdisciplines all have their specific cultures in how results are published and disseminated and target specific venues.

A challenge for interdisciplinary teams is therefore how to decide upon the best target for their work: should their work find place in a technical journal or a domain specific journal in order to reach the broadest possible audience? With increased availability of electronic sources, search engines are likely to return results from all sources, and hence findability is not an issue anymore. Perhaps more challenging is the culture of scientific recognition of publications, which differs widely between different disciplines. We support a holistic approach by disseminating interdisciplinary results as widely as possible and in all relevant communities. For this, information scientists could be encouraged to explore new possibilities and publish their novel methods in outlets of the target domain science, in this case movement ecology. Many of these methods have been developed for a purpose, and the ICT researchers have been able to benefit in the interdisciplinary collaborations from the data and expert knowledge contributed by the domain specialists. On the other hand, ecologists should be encouraged to collaboratively publish in ICT venues and follow these in order to benefit from the state-of-the-art in movement analysis in technical disciplines. This paper offers a useful list of ICT sources that are less familiar to ecologists, but worth exploring.

The above are some of the challenges that interdisciplinary collaborations in movement ecology may be facing. Of course, there are more challenges, particularly regarding technical issues or priorities of the research agenda. Some of these have already been addressed in the review of this paper. Challenges for the research agenda in movement research have also been sketched out in other initiatives that took place within the MOVE network, such as [224], who defined a research agenda focusing on the implications of working with real movement data, or [225] who defined the grand challenges of computational movement analysis. With new developments in tracking and other sensor technology, movement research is entering a golden age, with many more opportunities than challenges lying ahead. We believe that working across disciplines will allow researchers to address more and more ambitious questions about movement and we trust that MOVE has demonstrated this in a first attempt to raise interdisciplinary awareness. Further, we believe that interdisciplinarity can spark and foster unusual, innovative and exciting new ideas for movement research that no single discipline can produce on its own. We have experienced this within the MOVE network, as we hope this review demonstrates. We therefore also hope that the paper will achieve its goal of serving as a catalyst for further interdisciplinary collaborations in movement research.
